# Replacement of inorganic zinc with lower levels of organic zinc (zinc nicotinate) on performance, hematological and serum biochemical constituents, antioxidants status, and immune responses in rats

**DOI:** 10.14202/vetworld.2015.1156-1162

**Published:** 2015-09-30

**Authors:** D. Nagalakshmi, K. Sridhar, S. Parashuramulu

**Affiliations:** 1 Department of Animal Nutrition, College of Veterinary Science, Korutla, Karimnagar - 505 326, Telangana, India; 2 Department of Animal Nutrition, College of Veterinary Science, Hyderabad - 500 030, Telangana, India

**Keywords:** antioxidants status, hematological and serum biochemical constituents, immune responses, performance, rats, zinc nicotinate

## Abstract

**Aim:**

A study was undertaken to investigate the effect of organic zinc (zinc nicotinate, Zn-nic) supplementation (6, 9, and 12 ppm) compared to inorganic zinc (12 ppm) on growth performance, hematology, serum biochemical constituents oxidative stress, and immunity in weaned female Sprague–Dawley rats.

**Material and Methods:**

A 48 weaned rats (285.20±1.95 g) were randomly distributed to 4 dietary treatments with 6 replicates in each and reared in polypropylene cages for 10 weeks. Basal diet (BD) was formulated with purified ingredients without zinc (Zn). Four dietary treatments were prepared by adding 12 ppm Zn from ZnCO_3_ (control) and 6, 9, and 12 ppm Zn from Zn-nic to the BD. On 42^nd^ day, blood was collected by retro-orbital puncture for analyzing hematological constituents, glucose, cholesterol, alkaline phosphatase, total protein, albumin, and globulin and antioxidant enzyme activities. At 43^rd^ day, rats were antigenically challenged with sheep red blood cell (RBC) to assess humoral immune response and on 70^th^ day cell-mediated immune response.

**Results:**

Weekly body weight gains, daily feed intake, blood hematological constituents (white blood cell, RBC, hemoglobin concentration, packed cell volume, mean corpuscular volume, lymphocyte, monocyte, and granulocyte concentration) and serum glucose, total protein levels were comparable among the rats feed Zn from ZnCO_3_ and Zn-nic (6, 9, and 12 ppm). Serum cholesterol reduced with organic Zn supplementation at either concentration (6-12 ppm). Serum globulin concentration reduced (p<0.05) with 6 ppm Zn-nic supplementation compared to other dietary treatments. Lipid peroxidation lowered (p<0.05) reduced with 12 ppm organic Zn; thiobarbituric acid reacting substances and protein carbonyls concentrations in liver reduced (p<0.05) with 9 and 12 ppm levels of organic Zn supplementation compared to 12 ppm Zn supplementation from inorganic source. RBC catalase and glutathione peroxidase enzymes activities were highest (p<0.05) in rats supplemented with 12 ppm Zn-nic, followed by 9 ppm. Comparable immune response (humoral and cell-mediated) was observed between 12 ppm inorganic Zn and 9 ppm organic Zn and higher (p<0.05) immune response was noticed at 12 ppm Zn-nic supplementation.

**Conclusion:**

Based on the results, it is concluded that dietary Zn concentration can be reduced by 50% (6 ppm) as Zn nicotinate without affecting growth performance, hemato-biochemical constituents, antioxidant status, and immunity. In addition, replacement of 12 ppm inorganic Zn with 12 ppm organic Zn significantly improved antioxidant status and immune response.

## Introduction

Zinc (Zn) is component of more than 300 metalloenzymes and influences various biological functions [1]. In addition to that Zn is essential for humoral and cell-mediated immune (CMI) responses [2] and also plays a vital role in antioxidant defense system [3]. Therefore, Zn has significant therapeutic benefits in several diseases in humans and livestock.

Recently, concept of organic minerals come into picture in which mineral is in a chemically inert form, more stable and less prone to mineral and nutrient interactions, so absorbed and circulated to target tissues very efficiently [4]. Some researchers in mineral nutrition have shown that lower levels of zinc supplementation in organic form is sufficient to meet the requirements [5-7] of minerals. Many organic sources of Zn are available, i.e. Zn proteinate, Zn amino acid complex, Zn polysaccharide, Zn methionine, Zn glycinate, and their efficiency has been tested in many livestock species in terms of growth, immunity, and reproduction [8]. Zn nicotinate is an organic source of Zn with nicotinic acid (vitamin) as the ligand.

The literature on supplementing Zn-nic as a source of Zn in diets is no available. Hence, the present study was carried out to know the influence of replacement of inorganic zinc with graded levels zinc nicotinate (organic source) on performance, antioxidant enzymes, and immunity in rats.

## Materials and Methods

### Ethical approval

The experiment was approved by Institutional Animal Ethics Committee.

### Feeding and housing management

The 48 female Sprague–Dawley strain rats with an average body weight of 285.20±1.947 g were housed in polypropylene cages in the Animal House of College of Veterinary Science, Hyderabad. Rats were managed under hygienic conditions with controlled temperature (22-23°C) and photoperiod (12 h/d) for an experimental duration of 10 weeks. The rats were reared as per the guidelines of Institutional Animal Ethics Committee of the college. The rats were randomly distributed to 24 replicates with 2 rats in each and these 24 replicates further randomly allotted to 4 dietary treatments. The rats were offered respective diet at *ad libitum* level with the provision of free access to wholesome clean deionized water through polypropylene bottles having nipples. A control diet was prepared with purified ingredients but without Zn (Table-1) as per the formulae of AIN - 76A. The control diet supplied 12 ppm Zn through inorganic source of zinc (Zn carbonate). The three experimental diets contained Zn-nicotinate so as to supply zinc at concentration of 6, 9 and 12 ppm. Weekly body weights and daily feed intake were recorded. Blood was collected on 42^nd^ day by retro-orbital puncture to analyze the hematological and biochemical constituents and antioxidant enzymes. On 43^rd^ day of experiment, humoral immune response was examined by antigenically challenging the rats with sheep red blood cell (RBC). On 70^th^ day of experiment, the CMI response was assayed by footpad reaction method.

**Table-1:** Ingredient composition of purified diet (AIN-76A).

Ingredient	Proportion, g/kg diet
Sucrose	500.0
Casein	200.0
Corn starch	150.0
Oil	50.0
Cellulose	50.0
Mineral mixture*	35.0
Vitamin mixture*	10.0
DLmethionine	3.0
Choline chloride	2.0

* Mineral mixture and vitamin mixture was prepared as per specifications for AIN-76A

### Hematological and biochemical constituents

For hematology, blood was collected in heparinized vacutainers from all rats on 42^nd^ day. Hemoglobin (Hb) content, and white blood cell (WBC) counts, hematocrit, mean corpuscular volume, mean corpuscular Hb, lymphocyte, monocyte, and granulocyte percentages were determined by automatic blood analyzer (Huma Count, Med Source Ozone Biomedical Pvt., Ltd., India). Serum was collected and stored at −20°C in Eppendorf tubes for estimation of biochemical constituents, i.e. total protein [9], albumin [10], glucose [11], cholestrol [12], and alkaline phosphatase (ALP) [13].

### Oxidative stress markers and antioxidant enzyme activity

#### In hemolysate

The blood collected in clean heparinized vacutainers was centrifuged at 2000 rpm for 15 min at 4°C to separate buffy coat and erythrocyte pellet. The erythrocytes were washed thrice with phosphate buffer saline (pH 7.4). The packed RBC obtained was mixed with an equal volume of phosphate buffer saline and then diluted as per requirement with distilled water. The oxidative enzymes *viz*., RBC catalase (CAT), lipid peroxidation (LPx), glutathione peroxidase, (GPx) and glutathione reductase in hemolysate were estimated as per the procedures of Bergmeyer [14], Placer *et al*. [15], Paglia and Valantine [16] and Carlberg and Mannervik [17], respectively. The Hb and protein concentration in hemolysate were estimated colorimetrically as per the procedure described by Cannan [18] and Lowry *et al*. [19], respectively.

#### In liver

After 10 weeks of experiment, all rats were sacrificed and livers were collected, which were perfused with normal saline (0.9%) immediately to reduce red blood cell contamination. The samples were then fixed in liquid nitrogen and stored at −20°C for antioxidant analysis. The markers thiobarbituric acid reacting substances (TBARS), reduced glutathione (GSH), and protein carbonyls were estimated as per the procedures of Balasubramanian *et al*. [20], Moron *et al*. [21] and Levine *et al*. [22], respectively.

### Immune response

The rats were anti-genically challenged twice with sheep RBC (0.5 × 10^9^ cells/100 g, I/P) at 7 days interval to study the immune response. The blood was collected from retro-orbital plexus of all anti-genically challenged rats after 1 week of primary and secondary challenge to separate the serum. 25 μl of serum was serially diluted with 25 μl of phosphate-buffered saline (PBS). Sheep RBC (0.025 × 109 cells) were added to each of these dilutions and incubated at 37°C for 1 h. The rank of minimum dilution that exhibited hemagglutination was considered as the antibody titer. The CMI was assayed by footpad reaction method [23]. The increase in the paw volume measured after 48 h of inducing sheep RBC (0.025 × 10^9^ cells), in the subplantar region of the right hind paw. The mean percent increase in paw volume was considered as a delayed type hypersensitivity reaction and considered as an index of CMI. The volume of the left hind paw was injected similarly with PBS that served as control.

### Statistical analysis

The experimental results obtained were statistically analyzed using one-way ANOVA according to the method of Snedecor and Cochran [24]. The means were compared by Duncan’s multiple range test [25].

## Results and Discussion

### Performance

In the present study, body weight gains and feed intake were comparable among the dietary treatments (Table-2). Zinc is components of more than 300 enzymes, and it is essential for DNA synthesis, fetal development, brain development, normal growth, reproduction, membrane stability, bone formation, and wound healing [26]. Rossi *et al*. [27] stated that Zn deficiency or low levels of Zn supplementation causes depressed appetite, resulting in decreased feed intake and reduced weight gain. However, in present experiment reducing the dietary zinc supplementation up to 6 ppm using organic source (Zn-nic) did not affect the weekly weight (Table-2) and feed intake (Table-3) of the rats and was comparable with rats fed on diets supplemented with 12 ppm Zn as zinc carbonate. This might be due to higher bioavailability of Zn-nic (organic Zn) that could have reduced the amount of Zn supplementation required for body weight gain and efficiency. Our results were inconsistent with the findings of Feng *et al*. [6] who observed comparable average daily gains in broilers fed on dietary Zn supplementation of 90 mg Zn/kg diet from Zn-gly and 120 mg Zn/kg diet from ZnSO_4_. Similarly, Zn supplemented as Zn-met (250 mg/kg) resulted in comparable weight gains in pigs as with higher levels of ZnO (3000 mg/kg) [28].

**Table-2:** Body weight changes (g) in rats fed organic zinc supplemented diets at varied concentration.

Week	12-I	6-O	9-O	12-O	SEM	p value
Start	285.6	285.5	284.1	285.6	1.947	0.992
1	291.1	290.1	285.1	290.3	1.960	0.705
2	290.9	290.1	286.7	291.0	1.784	0.799
3	293.8	292.5	291.7	293.5	1.782	0.976
4	295.3	294.0	292.9	296.7	1.868	0.907
5	298.3	296.3	294.3	298.1	1.838	0.865
6	299.7	298.0	296.2	299.4	1.812	0.906
7	301.5	299.6	297.8	301.3	1.787	0.881
8	303.6	301.2	299.9	303.1	1.797	0.885
9	305.4	302.8	302.4	305.6	1.768	0.885
10	307.2	304.9	304.8	308.3	1.763	0.875

O=Organic Zn, I=Inorganic Zn, SEM=Standard error mean

**Table-3:** Daily feed intake (g) in rats fed organic zinc supplemented diets at varied concentration.

Week	12-I	6-O	9-O	12-O	SEM	p value
1	24.24	24.87	25.02	24.85	0.160	0.342
2	24.60	24.97	24.70	25.00	0.118	0.572
3	24.87	24.40	24.59	24.12	0.148	0.342
4	23.68	23.80	23.98	24.97	0.196	0.070
5	24.40	24.03	24.12	24.31	0.129	0.748
6	24.43	24.60	24.77	24.57	0.105	0.743
7	24.50	24.38	24.83	24.57	0.110	0.567
8	24.33	24.04	24.59	24.60	0.127	0.379
9	24.50	24.74	24.80	24.83	0.086	0.547
10	24.42	24.40	24.70	24.72	0.064	0.138
Av. intake	24.40	24.43	24.61	24.66	0.068	0.457

O=Organic Zn, I=Inorganic Zn, SEM=Standard error mean

### Hematological constituents

El Hendy *et al*. [29] reported that reduced Zn supplementation leads to significant (*P*<0.05) reduction in RBC count, Hb concentration and packed cell volume. However in the present study, different levels of zinc supplementation, either from ZnCO_3_ (12 ppm) or Zn-nic (6-12 ppm) had no effect on various hematological constituents analyzed (Table-4). Thymulin is a Zn dependent hormone which is essential for maturation of T lymphocytes [30], thus Zn deficiency cause apoptosis of lymphoid cells and leads to reduction in total leucocytic and lymphocyte count [31]. Similarly, Akbari *et al*. [32] reported that addition of 60 mg Zn/kg ZnO to basal diet (BD) significantly (p<0.05) increased WBC and lymphocyte count in broiler chicks. However, in the present study total WBC and lymphocyte count were comparable among all four dietary treatments. Organic trace minerals are stable in digestive tract and mineral would be protected from forming complexes with other dietary components that inhibit interactions and thus allow for great absorption [4]. This might be the reason for comparable WBC and lymphocyte count among all four dietary treatments.

**Table-4:** Hematological and biochemical constituents in rats fed organic zinc supplemented diets at varied concentration.

Attribute	12-I	6-O	9-O	12-O	SEM	p value
Hematological constituents						
Hb (%)	16.02	14.56	14.18	14.66	0.285	0.105
RBC (×10^6^/cu mm)	9.54	8.76	9.85	8.76	0.248	0.305
WBC (×10^3^/cu mm)	11.56	10.81	10.65	9.91	0.416	0.605
HCT (%)	65.04	57.54	56.52	57.88	1.307	0.070
MCV (%)	64.00	65.60	66.00	64.80	0.341	0.164
MCH (%)	16.22	16.56	16.36	16.42	0.129	0.848
Lymphocytes (%)	74.14	79.70	79.64	78.58	0.935	0.105
Monocytes (%)	5.78	4.20	4.02	4.26	0.550	0.676
Granulocytes (%)	18.94	15.86	15.88	17.12	0.660	0.319
Biochemical constituents						
Glucose, mg/dl	136.4	141.2	142.1	134.7	2.096	0.559
Cholesterol, mg/dl	75.50^a^	58.32^b^	60.04^b^	63.74^b^	2.130	0.009
ALP, IU/L	46.27	57.28	64.99	67.20	3.755	0.192
Total protein, g/dl	6.52	6.33	6.32	6.22	0.051	0.469
Albumin, g/dl	4.14^b^	4.42^a^	4.03^b^	4.07^b^	0.046	0.003
Globulin, g/dl	2.38^a^	1.90^b^	2.29^a^	2.37^a^	0.057	0.002

ab Means with different superscripts in a row differ significantly, p<0.01, SEM=Standard error mean, O=Organic Zn; I=Inorganic Zn, ALP=Alkaline phosphatase, Hb=Hemoglobin, RBC=Red blood cell, WBC=White blood cell, HCT=Hematocrit, MCV=Mean corpuscular volume, MCH=Mean corpuscular Hemoglobin

### Serum biochemical constituents

Serum total protein, glucose, and ALP activity (Table-5) in the present study was comparable among four dietary treatments. However, the ALP activity increased linearly (p>0.05) with gradual increase in level of organic Zn supplementation. Higher ALP activity of about 45.23% was noticed with 12 ppm Zn-nic supplementation compared to same level of Zn supplemented from the inorganic source. Zn being an integral component of ALP, higher bioavailability of organic source [33] might have resulted in increased ALP activity. Similarly, many workers [28,34] reported increase in ALP activity with Zn supplementation. Reducing Zn supplementation from 12 to 9 ppm from Zn-nic did not affect serum total protein, albumin and globulin concentration, but further reduction in Zn concentration (6 ppm) reduced globulin concentration (Table-5).

**Table-5:** Oxidative enzyme activities in hemolysate and liver in rats fed organic zinc supplemented diets at varied concentration.

Attribute	12-I	6-O	9-O	12-O	SEM	p value
In hemolysate						
LPx (nM MDA/g Hb)	38.30^ab^	45.40^a^	32.56^ab^	21.88^b^	2.346	0.047
RBC catalase (µmole/min/Hb)	8.04^ab^	5.14^b^	9.90^a^	10.98^a^	0.650	0.002
GPx (µmole/mg protein)	5.22^c^	2.77^c^	10.27^b^	15.29^a^	1.112	0.001
Glutathione reductase (µmole/mg protein)	13.37	12.06	10.76	13.03	1.276	0.903
In liver						
TBARS (nM MDA/mg protein)	0.0194^a^	0.0187^ab^	0.0133^b^	0.0132^b^	0.0011	0.047
Protein carbonyls (nM/mg protein)	0.947^ab^	0.993^a^	0.888^b^	0.883^b^	0.0161	0.044
Reduced glutathione (µM/mg protein)	12.30^a^	9.21^b^	11.11^ab^	13.60^a^	0.518	0.016

abc Means with different superscripts in a row differ significantly: p<0.05, p<0.01; SEM: Standard error mean, O=Organic Zn, I=Inorganic Zn, LPx=Lipid peroxidation, GPx=Glutathione peroxidase, MDA=Malondialdehyde, RBC=Red blood cell, Hb=Hemoglobin, TBARS=Thiobarbituric acid reacting substances,

The anti-atherogenic effect of Zn was reported by Beattie *et al*. [35]. Zinc deficiency causes increased plasma lipid levels and an increased risk of cardiovascular diseases in low-density lipoprotein receptor knock-out mice and mice with Zn-deficient diets exhibited increased cholesterol and triglycerides levels in blood plasma [36]. Bolkent *et al*. [37] proved the protective effect of Zn supplementation on lipid metabolism indices (total lipids, cholesterol, high density lipoprotein cholesterol) in laboratory rats with streptozotocin-induced Type 1 diabetes. In the present study serum cholesterol concentration was reduced by organic Zn supplementation (Table-5) compared to inorganic Zn supplementation. This observation was supported by Parák and Straková [38] who stated that Zn supplementation could reduce the blood plasma cholesterol concentration, and the reduction was significantly higher with organic Zn supplementation compared to inorganic. Similarly, Sahin *et al*. [39] observed decrease in serum cholesterol concentrations in heat stressed Japanese quails by addition of 30 or 60 mg Zn/kg BD either from organic (zinc picolinate) or inorganic (ZnSO_4_) source, and the decrease was comparatively higher with organic Zn.

### Antioxidant status

LPx is a free radical mediated chain reaction, considered as best marker of oxidative stress [40] since its concentration increases during oxidative stress. Though LPx is a self-perpetuating reaction, its propagation can be arrested by chain breaking antioxidant. Zn plays an important role in the antioxidant system in two ways: The first is the protection of proteins and enzymes against free radical attack or oxidation [41], the second is through the prevention of free radical formation by other metals, such as iron and copper [42]. In liver TBARS and protein carbonyl concentration are indicative of oxidative stress and these markers significantly (p<0.05) lowered (Table-5) with Zn-nic supplementation at 9 or 12 ppm level compared to other dietary treatments and almost a similar trend was observed for LPx in haemolysate. GPx and CAT are involved in the antioxidant defense system and protects from potential oxidative damage [43,44]. In this experiment supplementation of 12 ppm Zn as Zn-nic significantly (p<0.05) increased the activities of CAT, GPx and lowered the LPx (malondialdehyde concentration) (Table-5) compared to others. Similarly, GSH concentration in liver (Table-5) was significantly (p<0.05) lower at 6 ppm Zn as Zn-nic and increased gradually with an increase in the level of Zn supplementation irrespective of source. The antioxidant enzymes activity and LPx was comparable between rats fed with 6 ppm Zn as Zn-nic or 12 ppm Zn as ZnCO_3_ in both liver and hemolysate clearly indicating Zn requirement could be reduced supplementation by 50% if supplemented from organic source. Bun *et al*. [45] observed improvement (p<0.01) in GPx activity with Zn supplementation in dose-dependent manner in broilers (0, 20, 40, and 60 ppm). Reducing the Zn supplementation from 12 to 9 ppm, as Zn-nic, still had higher activities of GP_X_ (p<0.01) and CAT (by 23.13%) than those with 12 ppm Zn supplementation from inorganic source, which could be due to higher bioavailability of Zn from Zn-nic (organic source) than ZnCO_3_. Our results were in similar to the findings of Ma *et al*. [46] who observed improvement in activities of superoxide dismutase and GPx enzymes in liver with 90 mg Zn/kg diet supplementation as Zn-gly compared to broiler chicks fed 120 mg Zn/kg diet as ZnSO_4._

### Immunity

Several researchers observed better immune response with increasing the level of Zn supplementation [47-50]. Zn is essential for maintenance of natural killer cells activity and phagocytosis of macrophages and neutrophils [2]. In the present study, the humoral and CMI response was higher (p<0.05) with 12 Zn supplemented as Zn-nic than ZnSO_4_, indicating the beneficial effect of supplementing Zn from organic source (Table-6). The humoral and CMI response was not affected by reducing the amount of Zn supplementation by 50-75% (6 and 9 ppm) when supplemented as Zn-nic that was comparable to that of 12 ppm supplementation from inorganic source (ZnCO_3_). Higher Zn availability with organic Zn resulting in higher antioxidative status in rats could have protected the immune cells from damage of free radicals and improved the activity of immune cells [33,41]. Similar to present study findings, Feng *et al*. [7] noticed (p<0.05) better immune response with supplementation of 90 mg Zn/kg as Zn-gly compared to 120 mg Zn/kg as ZnSO_4._ Hudson *et al*. [51] observed higher cellular immune response to PHA-P (p<0.05) and antibody titers against New castle disease in broiler breeders fed diets supplemented with 160 ppm Zn from Zn acetate than from ZnSO_4_. Similarly, Moghaddam and Jahanian [5] observed better immune response by replacing 50% of ZnSO_4_ (inorganic) with Zn-met (organic) in diets of broilers containing 40 ppm Zn as ZnSO_4_.

**Table-6:** Humoral and cell mediated immune response in rats fed organic zinc supplemented diets at varied concentration.

Attribute	12-I	6-O	9-O	12-O	SEM	p value
Humoral immune response, HA (log_2_ titers)						
Primary response	5.00	4.20	4.40	5.00	0.176	0.256
Secondary response	6.00^b^	6.60^b^	6.60^b^	7.60^a^	0.188	0.014
CIM response (% increase in paw volume)	4.88^ab^	3.43^b^	4.09^ab^	5.55^a^	0.285	0.043

ab Means with different superscripts in a row differ significantly: p<0.05, O=Organic Zn, I=Inorganic Zn, SEM=Standard error of mean, CIM=Cell mediated immune

## Conclusion

Based on the results, it could be concluded that dietary Zn concentration can be reduced by 50% (6 ppm) as Zn nicotinate without affecting growth performance, hematological and serum biochemical constituents, antioxidant status, and immunity. In addition, replacement of 12 ppm inorganic Zn with 12 ppm organic Zn significantly improved the antioxidant status and immune response.

## Authors’ Contributions

DNL planned the experiment and implemented the study design. KS and SPR recorded the data and analyzed the samples under supervision of DNL. DNL, KS, and SPR drafted the manuscript. DNL, KS, and SPR revised the manuscript. All authors read and approved the final manuscript.
